# Light-triggered drug release via fiber optic heater-integrated with thermoresponsive microgels for locoregional cancer therapy

**DOI:** 10.1038/s41598-026-48134-w

**Published:** 2026-04-27

**Authors:** Tania Mariastella Caputo, Gaia Maria Berruti, Silvia Vanni, Angela Maria Cusano, Claudia Cocchi, Chiara Liverani, Marco Consales, Laura Mercatali, Toni Ibrahim, Alessandro De Vita, Anna Aliberti, Andrea Cusano

**Affiliations:** 1 Optoelectronics Group, Department of Engineering, University of Sannio, Palazzo dell’Aquila-Bosco Lucarelli, C.so Garibaldi 107, 82100 Benevento, BN Italy; 2 Preclinic and Osteoncology Unit, Bioscience Laboratory, IRCCS Istituto Romagnolo per lo Studio dei Tumori (IRST) “Dino Amadori”, Via P. Maroncelli 40, 47014 Meldola, FC Italy; 3 CeRICT scrl Regional Center Information Communication Technology, Benevento, Italy; 4 Osteoncology, Bone and Soft Tissue Sarcomas and Innovative Therapies Unit, IRCCS Istituto Ortopedico Rizzoli, Bologna, Italy

**Keywords:** Optical fiber heater, Thermoresponsive microgels, Locoregional drug delivery, Breast cancer, Tumor therapy, Cancer, Chemistry, Drug discovery

## Abstract

Cancer treatment faces significant challenges due to the nonspecific delivery of chemotherapeutic drugs, resulting in systemic toxicity and limited efficacy. Targeted, localized drug delivery systems offer a transformative approach to overcome these limitations by improving therapeutic precision and minimizing side effects. Here, we propose the development of a novel light-to-heat triggered drug delivery (FO-LTDR) platform by integrating a fiber optic heater (FOH) with doxorubicin-loaded thermoresponsive microgels (DOX@MGs) for the locoregional treatment of cancer. The DOX@MGs, developed using a N-isopropylmethacrylamide-maleic acid formulation, exhibit superior drug loading capacity and controlled release properties, that ensure stability under physiological conditions and selective activation under external thermal stimuli. The FOH is engineered with a core-offset fusion splice and a gold-coated region that enables efficient conversion of optical to thermal energy. The thermal response of the platform is monitored in real time via an integrated fiber Bragg grating. The heat generated by the fiber results in a “squeezing effect” of the microgels covalently bound to its surface, releasing pure doxorubicin in a controlled manner strictly at the site of interest. The FO-LTDR platform demonstrated precise, on-demand drug delivery in 2D/3D MCF7 models, providing a versatile and controllable approach that could transform future strategies for localized chemotherapy.

**Supplementary Information:**

The online version contains supplementary material available at 10.1038/s41598-026-48134-w.

## Introduction

Cancer is a complex disease marked by uncontrolled cell growth, tissue invasion, and metastasis, driven by genetic mutations and disrupted cellular regulation. Standard intravenous chemotherapies often lack specificity, causing off-target toxicity and patient suffering^1,2^. Even when drugs reach the tumor, penetration is hindered by the altered tumor microenvironment (TME)^3^, necessitating high systemic doses that further exacerbate side effects. Achieving precise, targeted interactions between drugs and cancer cells is therefore critical for improving outcomes^4^. Advances in anticancer therapies have aimed to enhance chemical specificity, either by increasing cytotoxicity toward cancer cells or minimizing effects on healthy cells^5,6^. One promising strategy is stimuli-assisted, on-demand drug delivery^7,8^, where drugs are encapsulated in carriers—such as liposomes^9–14^, porous polymers^15–17^, protein based^18^ or inorganic nanoparticles^19–21^ and released in targeted regions in response to an external stimulus^22–26^. Among them, Microgels (MGs) have garnered significant interest in the pharmaceutical^27^, biomedical, and biomaterial fields^28^ due to their versatile properties. These spherical, three-dimensional hydrophilic polymer networks range in size from a few nanometers to several micrometers and are recognized for their mechanical strength. Moreover, MGs can be synthesized with tailored chemical compositions to achieve biocompatibility and stimuli-responsiveness features that are critical for biomedical applications^29,30^. Interestingly, MGs are particularly well-suited for developing on-demand drug delivery systems, as they can modify their chemical-physical properties in response to stimuli^31^ (i.e. pH, temperature, ionic strength, magnetic, electrical, or chemical signals). This feature enables to load drugs into their highly porous structure and locally release them in a controlled and tuneable manner upon receiving specific stimuli. Advancing optimal drug delivery also requires controlling the physical localization of anticancer agents, enhancing specificity by restricting exposure to tumor regions. Localized delivery combined with controlled release represents a paradigm shift from systemic therapies, offering minimal invasiveness, precise targeting, and reduced off-target effects^32–34^. These strategies can be grouped into two categories: direct administration of conventional chemotherapeutics to the tumor site, and integration with advanced delivery platforms to improve targeting and efficacy. Although well-established and advantageous over conventional methods, localized chemotherapy is mostly limited to accessible malignancies treatable via minimally invasive, extracorporeal techniques^35^. In this context, optical fiber-assisted drug delivery technology, employing light as a release stimulus, represents a promising pathway to overcome these limitations due to its small size, which allows for integration into medical needles, catheters and miniaturised nano-endoscopes ensuring biocompatibility, miniaturization and cost-effectiveness at the same time. Such features make optical fiber-based devices a strategic compromise for light-activated locoregional drug delivery: while moderately invasive than systemic administration, they enable precise, site-specific treatment within the target organ, thereby maximizing therapeutic efficacy and minimizing off-target effects^36^. Although the use of optical fibers as light-assisted platforms for localized clinical treatments − such as laser surgery, photothermal (PTT), and photodynamic (PDT) therapies^37–43^—is well established, only a small number of studies^44–46^ have investigated their potential as dual-function platforms, serving both as ‘drug transporters’ to defined spatial sites and as triggers for cargo release upon appropriate optical stimulation.

Nazari and colleagues^44^ developed a drug delivery platform in which an optical fiber is coated with a thin UiO-66 metal–organic framework loaded with 5-fluorouracil (5-FU). Light triggers 5-FU release via the fiber, enabling precise cancer treatment; however, the system’s payload was limited, requiring ~ 180 fibers to reach therapeutic doses. Building on this, we conducted a feasibility study using a light-triggered system based on side-emitting optical fibers (seOF) integrated with Sorafenib-loaded PLGA particles via a UV photocleavable linker^45^. Sorafenib, a small hydrophobic molecule with limited biodistribution and rapid metabolism, was encapsulated in FDA-approved PLGA, immobilized on seOF with a photocrosslinker, and tested in a microfluidic device. This enabled precise locoregional delivery, enhanced cellular uptake, and improved cytotoxic efficacy, potentially reducing systemic side effects. Here, drug release relies on light cleaving the photo-crosslinkers along the fiber. Emerging approaches in drug delivery have introduced indirect light-mediated release as a strategy to address limitations in payload, carrier immobilization, and energy use. Such innovative technique integrate optical fibers with “smart” materials that respond to environmental stimuli, such as temperature changes, altering their conformation or physicochemical properties, offering novel platforms for precise light-controlled drug delivery^47^. In a study by Zhang et al.^46^ an optical fiber-based method for photothermal-chemotherapy used heat to break down doxorubicin-loaded agarose clots. While therapeutically effective, this platform struggles with dose standardization. The size variability of the clots compromises reproducible cellular uptake, and the simultaneous release of agarose^48,49^ introduces potential toxicity and clearance issues, hindering consistent long-term delivery.

In this context, we developed a novel nanophotonic platform for loco-regional, “light-to-heat triggered doxorubicin (DOX) release” (LTDR). It relies on the covalent integration of DOX-loaded thermoresponsive microgels (DOX@MGs) along the lateral surface of an engineered fiber optic heater (FOH)^50^. DOX, a well-established anthracycline chemotherapeutic^51^, interferes with DNA/RNA synthesis and inhibits topoisomerase II, triggering apoptosis^52^. Its clinical use, however, is limited by severe side effects^53^, particularly cardiotoxicity^54^, due to lack of tumor specificity and the high systemic doses required to reach therapeutic concentrations in target tissues. Herein, thermoresponsive DOX@MGs were designed and judiciously developed to obtain high encapsulation efficiency (EE) and superior drug-loading (DL) capacity, attributed to the innovative N-isopropylmethacrylamide (NIPMAM)–maleic acid formulation. The optimized thermoresponsive properties (volume phase temperature transition above physiological temperature conditions) ensure stable drug retention under physiological conditions and controlled release under specific external stimuli. Covalent immobilization of DOX@MGs on the fiber surface enables precise, site-specific drug delivery under light-mediated stimulation, thereby bypassing systemic administration and avoiding the release of carrier materials that could hinder clinical translation.

**Scheme 1:**
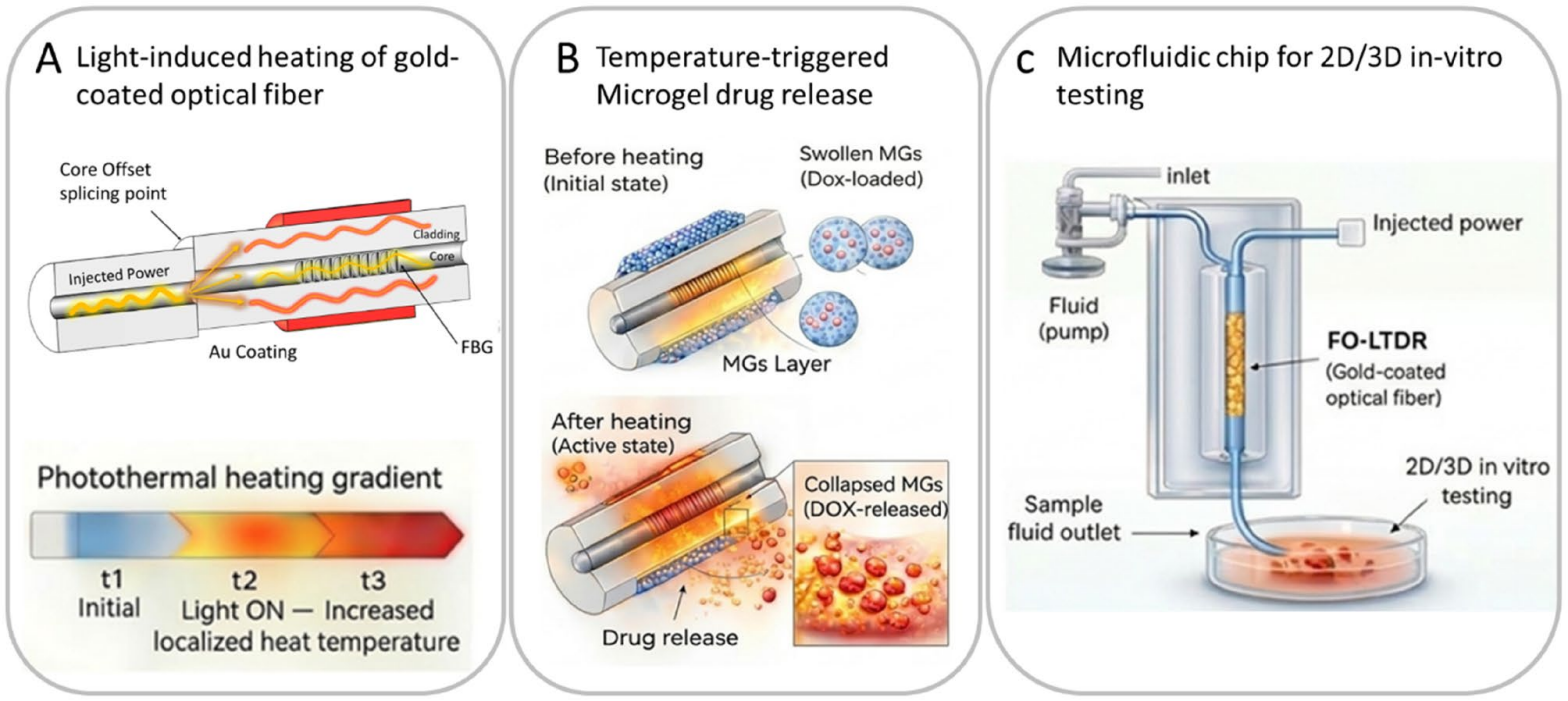
Overview of the FO-LTDR platform for light-triggered drug release and in vitro testing. (**A**) Light injected into the gold-coated optical fiber (FOH) generates a homogeneous photothermal effect along the gold-coated fiber surface. The temperature map illustrates the temporal evolution of the heating process from the initial state (t1) to full activation (t3) upon light exposure. (**B**) DOX-loaded microgels (MGs) are covalently immobilized onto the lateral gold-coated surface of the FOH. In the inactive state, the swollen MGs retain the drug within their network; upon light activation, the localised temperature increase triggers MGs collapse and controlled DOX release. (**C**) The FO-LTDR device is integrated into a microfluidic setup enabling both 2D and 3D in vitro testing: fluid is delivered by a pump through the inlet, flows past the laterally coated fiber surface, and the drug-containing effluent is collected at the outlet for direct application to cell culture systems.

As schematically represented in Scheme 1, the FOH consists of a core-offset fusion splice between two standard Single Mode (SM) fibers, one of which has at its termination a metal-coated region whose length can be tailored to suit the stable integration of the thermo-responsive carriers. Thanks to the core-offset junction, the light injected into the fiber core is efficiently transferred into the cladding region, where it can propagate towards the gold-coated active region, enabling the conversion of optical to thermal energy mediated by resistive heating through the metallic coating^50^. The platform is endowed with real-time thermal monitoring capabilities, achieved by inscribing a fiber Bragg grating (FBG) within the core of the coated fiber. Gold (Au) was selected as the coating material due to its advantageous physical and chemical properties, which make it highly suitable for the application under investigation. Specifically, thanks to its high absorption coefficient, Au exhibits excellent energy conversion efficiency, which can be even further optimized through plasmonic resonance phenomena, allowing the use of very small coating thicknesses (i.e., low thermal masses) that fasten the heater thermal response. Additionally, the gold’s high thermal conductivity allows for an efficient distribution of the heat generated along the fiber, thereby mitigating the risk of localized heat accumulation, and at the same time facilitates the immobilization of the carriers on its surface. The heat produced by the FOH is transferred to the smart DOX@MGs layer, which is covalently immobilized on the gold surface, promoting the MGs collapse according to the “squeezing effect” in a precise and tunable manner, thus enabling the release of pure DOX. The length of the Au-coated region represents, therefore, a degree of freedom of the proposed platform and can be used to match the drug payload required by the specific application. In addition, this method prevents the leakage of other materials or excipients, which could alter the pharmacokinetics of the drug, and facilitates a consistent and controlled drug release.

The performance of our fiber optic platform for light-to-heat triggered drug-release has been validated in vitro on the breast cancer cell lines (MCF7), both in 2D and 3D culture models, demonstrating robustness and effectiveness for locoregional drug delivery.

These results validate the FO-LTDR platform as a streamlined, cost-effective approach to breast cancer treatment^55^, overcoming limitations in manufacturing complexity, cost, and spatial/temporal control. Its precise, light-triggered drug release mechanism makes it a promising candidate for preclinical translation and a compelling solution for targeted therapy. Furthermore, taking into account the intrinsic light-to-heat conversion principle of the proposed device, the FO-LTDR platform could also be investigated for PTT applications. This dual functionality further strengthens its therapeutic significance, providing a unique and innovative strategy that can be simultaneously applied for both localized drug release and thermally induced tumor cell ablation.

## Experimental section

### Materials

N-isopropylmethacrylamide 97% (NIPMAM), N-isopropylacrylamide 97% (NIPAM), potassium persulfate (KPS, 98%), Maleic acid (MAAC), Acetonitrile (ACN), acetic acid, trifluoroacetic acid (TFA), Rhodamine 6G (99%), N-(3-dimetilaminopropil)-N′-etilcarbodiimide (EDC), Sodium 2-Morpholinoethanesulfonate (MES), Cysteamine, Doxorubicin hydrochloride, Sodium chloride, Potassium chloride, Phosphate buffered saline tablet and dialysis bag (cut off 12 kDa) were purchased from Sigma Aldrich. Deionized water was obtained from a Milli-Q Plus system from Millipore with a resistance of 18.2 MΩ cm and a total organic content (TOC) < 10 ppb.

### MGs synthesis

MGs (labelled DD1, DD2 and DD3) were prepared by free radical copolymerization of thermo-responsive NIPMAM and NIPAM monomers, MAAC, and N′-N′-methylenebisacrylamide (BIS), using KPS as initiator. In details, the syntheses were carried on a three-neck round-bottom flask equipped with a reflux condenser, a nitrogen inlet and a temperature probe, immersed in an in a silicon filled oil bath.

- For the synthesis of MGs DD1 Nipam and BIS solubilized in 98 mL of Milli-Q water were gradually brought up to 70 °C. The solution was continuously stirred at 500 rpm and purged with N_2_. The initiator KPS, previously solubilized in 1 mL of water, was added to the reaction flask to start the polymerization. After 15 min MAAC solubilized in 1 mL was added and the reaction mixture was allowed to proceed 4 h at constant stirring under N_2_ flow at 70 °C.
- MGs DD2 were obtained starting from the Nipmam, MAAC and BIS mixed in 96 mL of water. The solution temperature was brought up to 70 °C and was purged with N_2,_ under continuous stirring at 500 rpm. After 1 h of constant heating at 70 °C, the initiator KPS, previously solubilized in 4 mL of water, was added to the reaction flask. The polymerization was allowed to proceed 5 h at constant stirring under N_2_ flow at 70 °C.
- MGs DD3 were synthetized mixing Nipam, MAAC and BIS in 90 mL of water. The solution temperature was brought up to 70 °C and was purged with N2, under continuous stirring at 500 rpm. After 1 h of constant heating at 70 °C, the initiator KPS, previously solubilized in 10 mL of water, was added to the reaction flask. The polymerization was allowed to proceed 5 h at constant stirring under N2 flow at 70 °C.

All the resulting copolymerized microgels (See *Table S1* for MGs monomers quantitative and time of reaction used for each formulation) were purified by dialysis for 1 week against MilliQ water at 4 °C and lyophilized.

### Dynamic light scattering (DLS) measurement

The thermo-responsivity of MGs was investigated by DLS using a Zetasizer Nano ZS instrument (Malvern) equipped with a 633 nm laser (scattering angle 173°) and with a temperature controller. Freeze dried MGs were dispersed in Phosphate Buffered Saline (PBS) (5 mg/mL) and incubated overnight at 5 °C. Measurements were carried on 500 µL samples at 0.25 mg/mL. The temperature was raised from 25° to 55 °C with steps of 3 °C; an equilibration time of 1800s was used for each temperature and a total of 5 run were conducted. The experimental uncertainties represent the standard error of the mean of the replicate runs.

### Morphological analysis by atomic force microscopy (AFM)

Morphological characterizations of the MGs were directly performed onto glass slice covered by an Au layer by using an Agilent 5420 AFM system (Agilent Technologies, Santa Clara, CA, USA). AFM images were obtained by scanning the MGs film in tapping mode in order to avoid damage of the gel particles. All the measurements were carried out with the MGs in the dry state. The raw data collected by the AFM were processed with the Pico Image software (Keysight Technologies, Santa Rosa, CA, USA).

### Fourier transform infrared spectroscopy (FTIR) analysis

The MGs (both naked and DOX-loaded) and the DOX free were analysed by using a Perkin-Elmer Spectrum 3 infrared spectrometer (Perkin-Elmer, Waltham, MA, USA) equipped with an attenuated total reflectance (UATR) accessory. Spectra were acquired by 8 scans with a resolution of 2 cm^−1^ in the range 650–4000 cm^−1^, and a series of three replicates of each formulation was analysed.

### Morphological analysis by confocal laser scan microscopy (CLSM)

MGs were analysed by confocal laser scan microscopy (STELLARIS 8 -Leica Microsystems). A white light laser tunable between 440 and 790 nm was used for excitation and the samples were focused with a HC PL APO CS2 63x/1.10 oil immersion objective and Emission signals were acquired using Power HyD detectors, while, bright-field image were acquired with a PMT detector. For each sample, 30 µL of MGs solution 0.1 mg/mL in triplicate were collected into a microchannel of 6 channels ibidi (μ-Slide VI 0.4) and the excitation wavelength and detection window were respectively 482 nm and 496 to 600 nm. The system was controlled using Leica Application Suite (LAS) X v4.3 software.

### Encapsulation efficiency and drug loading

The encapsulation efficiency of DOX was evaluated by mixing freeze-dried MGs with DOX solutions of varying concentrations. A stock solution of DOX hydrochloride was first prepared by dissolving the drug in deionized water to a concentration of 10 mg/mL. From this stock, three working solutions were prepared in phosphate-buffered saline (PBS at pH 7.4) at final concentrations of 0.25 mg/mL, 0.5 mg/mL, and 2 mg/mL. One milliliter (1 mL) of each DOX working solution was then mixed with 1 mg of freeze-dried MGs. These mixtures were incubated overnight at 4 °C to allow for drug encapsulation. Following incubation, the samples were centrifuged at 16,000 rcf for 50 min at 4 °C to separate the drug-loaded microgels from any free, not encapsulated DOX. The resulting pellet, containing the drug-loaded MGs, was washed three times and then freeze-dried for subsequent drug release tests. The supernatant, containing the not encapsulated DOX, was collected and analyzed to determine the encapsulation efficiency (EE) and drug loading (DL). The concentration of the free drug was quantified via spectrophotometric analysis using a NanoDrop™ One (Thermo Fisher Scientific Inc.), measuring the absorbance intensity at 484 nm. A DOX calibration curve, established within the linear range of 0.125 µg/mL to 1 µg/mL, was used to quantify the amount of encapsulated drug.

In particular, the EE was calculated as:

$$EE(\% ) = \frac{(Ci - Cf)}{{Ci}}*100$$

where *C*_*i*_ is the initial concentration of DOX and *C*_*f*_ is the concentration of free DOX recovered in the supernatant after the MGs centrifugation.

The Drug Loading (DL) was calculated as:

$$DL(\% ) = \frac{(We)}{{Wp}}*100$$

where We is the weight of the encapsulated DOX, quantified by spectrophotometric analysis, and Wp is the weight of freeze dried MGs, before encapsulation procedure, used in the experiment.

### Drug release studies

The in vitro release of doxorubicin (DOX) from the microgels (DOX@MGs) was assessed using a dialysis bag method under four different conditions. For each condition, 1 mg of DOX@MGs was placed into a microdialysis membrane (Pierce 96-well Microdialysis Plates, 10 MWCO; Thermo Fisher Scientific Inc.). The membranes were then incubated in 1 mL of either physiological (phosphate-buffered saline—PBS at pH 7.4) or an acidic buffer^56,57^ (137 mM NaCl, 2.7 mM KCl at pH 4.0). The four tested conditions were: (i) physiological buffer at 37 °C; (ii) physiological buffer at 55 °C; (iii) acidic buffer at 37 °C; and (iv) acidic buffer at 55 °C. At predetermined time points, the entire buffer was collected and replaced with 1 mL of fresh, pre-warmed buffer to maintain sink conditions. The amount of DOX released into the buffer was quantified by measuring its fluorescence intensity. An EnSight Multimode Plate Reader (PerkinElmer, Waltham, MA, USA) was used with an excitation wavelength of 484 nm and an emission wavelength of 595 nm. The measured fluorescence intensity was then converted to DOX concentration using a calibration curve established in triplicate for both the physiological and acidic buffers.

### Design, fabrication and characterization FOH

The heating device at the basis of the proposed platform was entirely implemented in a standard single-mode optical fiber (acrylate SMF28e with a core diameter of 8.2 µm, a cladding diameter of 125.0 ± 0.7 µm and a coating diameter of 245.0 ± 5 µm). A 6 µm core offset junction (*Figure S1*)—performed with a commercially available splicing machine (Fujikura80S) – was previously demonstrated to result in an improved light coupling efficiency towards cladding compared to results reported in literature^50^.

The percentage of light coupled into the cladding after the optical junction was ~ 90%. This was measured by connecting the optical fiber probe directly to an optical power meter (ML9001A Anritsu) and evaluating the ratio between the output power and the power injected into the device. The core-offset was fabricated 2.5 mm away from the gold coating. A 5 mm long commercial Fiber Bragg Grating (FBG), with 75% of reflection and centered in the wavelength range between 1510 and 1590 nm, was integrated into the coated area. A 150 nm thick Au coating, extending over a length of 10 mm or 20 mm, was deposited on the fiber surface by vacuum evaporation in two steps, rotating the fiber by 180 °C at each run. Recent studies conducted by our research group demonstrated that a further increase in coating thickness and/or length leads to an undesirable increase in thermal mass, which in turn has a detrimental effect on the device heating efficiency^50^.

Each fabricated device was preliminarily cycled in an industrial climatic chamber (HPP110 from Memmert) in the range of 5–50 °C. The experimental setup equipped for the device characterization included a pump laser diode (Arroyo Instrument 6340 ComboSource) operating at 1485 nm and a commercial FBG interrogation system (Micron Optics sm125) featuring 80 nm wavelength bandwidth between 1510 and 1590 nm and a resolution of 1 pm, connected via a 1480/1550 nm Wavelength Division Multiplexer (WDM) (Thorlabs WD1450A). The optical probes were connected to the WDM channel operating at 1550 nm and their response was acquired through the ENLIGHT sensing analysis software, included with the sensing interrogation system throughout the experiment.

### MGs integration onto the fiber optic platform

To immobilize MGs on the gold surface of the probes, MGs were firstly functionalized with Cisteamine 0.1%w/v in presence EDC 0.5 M in 0.1 M MES buffer pH 4.8 overnight at room temperature. MGs were then washed three times with milliQ water and incubated with DOX (2 mg/mL) as described in the Encapsulation efficiency and Drug Loading paragraph*.* The fiber probe was dipped into 500 µL of a DOX loaded MGs solution at concentration of 3.75% w/v.by means of a dip coater (KSV NIMA KN4001, Biolin Scientific Oy) at a controlled speed of 0.5 mm/min and environmental temperature. The MGs film was then washed with PBS solution three times and the layer regularity qualified via CLSM (Figure S2).

### FO-LTDR for in vitro drug release

During the in vitro drug delivery experiments, the fiber optic devices were mounted vertically on a custom-designed support and immersed in buffer solution (400 µL and 500 µL for probes with lengths of 10 mm and 20 mm, respectively) using a dip coater (KSV NIMA KN4001, Biolin Scientific Oy) operating at a controlled speed of 5 mm/min and room temperature. Each activation test involved two identical optical probes fabricated according to the same design: one probe (hereafter referred to as “Activated”) was connected to the pump laser, while the other (designated as “Not Activated”) was left disconnected and used as a reference in the evaluation of the drug release performance. The pump laser was operated at fixed time intervals with an input power of 214 mW. Once the temperature along the Au layer of the Activated probe reached ~ 55 °C, the input power was slightly adjusted to maintain it stable at this value, thereby enabling light-induced drug release. The total activation time was 120 min. The typical response of the proposed platform with *l* = 10 mm during an internal activation test is shown in *Figure S3*, demonstrating the capability of the optical device to reach the VPTT within a few minutes. After each power injection, the solutions in which the two probes remained immersed were collected for fluorescence-based drug release analysis, as previously described, and replaced with fresh buffer at room temperature.

### Collagen scaffold preparation

The tridimensional collagen-based scaffold culture systems were synthesized in our laboratory as follows: a 1% (w/v) suspension of bovine-derived insoluble type I microfibrillar collagen was first dispersed in 0.05 M acetic acid. To this mixture, 1 M sodium hydroxide was added, followed by cross-linking with 1 wt% 1,4-butanediol diglycidyl ether (BDDGE) to stabilize the collagen network and modulate its porosity and tortuosity. The resulting suspension was homogenized using an IKA T18 Basic ULTRA-TURRAX and centrifuged to eliminate air bubbles. A controlled freezing and heating process (ranging from 25 °C to − 25 °C, then back to 25 °C over 50 min under vacuum at 0.20 mbar) was employed to achieve the desired porosity, ensuring optimal pore size, interconnectivity, and orientation. The scaffolds were then sterilized by immersing in 70% ethanol for one hour, followed by three washes with sterile Dulbecco’s Phosphate-Buffered Saline (Life Technologies, Carlsbad, CA, USA). Scaffold porosity and pore size were measured according to previously established methods^58,59^. In detail, the scaffold is highly porous (85.6%), meaning that most of its volume consists of empty space, as expected for tissue engineering materials. The void space (~ 50.1 × 10^3^ µm^3^) is much larger than the collagen volume (~ 9.1 × 10^3^ µm^3^), thereby enhancing permeability. In addition, the pore walls are relatively thick (~ 15.2 µm), which ensures mechanical stability despite the high porosity. All chemicals used were obtained from Sigma-Aldrich (St. Louis, MO, USA).

### Cell seeding and culture

The MCF7 human breast cancer cell lines (Research Resource Identifiers (RRIDs): CVCL_0031) were sourced from the American Type Culture Collection (Rockville, MD, USA). Cells were cultured in DMEM medium supplemented with 10% fetal bovine serum, 1% penicillin–streptomycin, and 1% glutamine (PAA, Piscataway, NJ, USA) at 37 °C in a 5% CO_2_ environment. For monolayer cultures, 2.5 × 10^5^ cells were plated in 6-well plates or 1 × 10^4^ cells were plated in 96-well plates. For 3D cultures, 5 × 10^5^ cells in 15 µL of medium were applied to the surface of each scaffold (1 × 5 mm) in a 24-well plate. Before seeding, scaffolds were dried to remove PBS using sterile tips. For the 3D culture, 1 × 10^5^ cells were resuspended in 15 µL of DMEM-H and seeded onto the scaffold placed in a well of a 24-well plate. After a 2-h incubation at 37 °C to promote adhesion, 2 mL of culture medium were added to the well.

### Drug treatment

DOX treatment was applied to both monolayer (2D) and 3D scaffold cultures at concentrations ranging from the human plasma peak (2.4 µg/mL) to 0.15 µg/mL by adding the drug in culture medium. Saline buffer (NaCl 137 mM, KCl 2.7 mM, pH 4) was used as a vehicle to dilute free DOX before administering it to the cells and as a control, to ensure that it does not alter drug properties and is not toxic to the cells. Cells in 6-well plates, 96-well plates and 3D collagen scaffolds were treated with the drug or vehicle the day after seeding. After 24 h of treatment, the culture medium was refreshed to remove any unbound drug. The DOX dosage was based on clinical pharmacokinetic data extrapolation and adjusted for local treatment applications.

### MTT assay for cell viability

Cell viability was assessed using the Thiazolyl blue tetrazolium bromide (MTT, Sigma-Aldrich) reduction assay. After 72 h of drug exposure, cells were incubated with 0.5 mg/mL MTT solution in DMEM for 2 h at 37 °C. Acidic isopropanol was prepared adding 1% of 10 M HCl to isopropanol. After treatment, cell cultures were incubated with 1 ml of acidic isopropanol under shaking conditions for 1 h to allow proper formazan solubilisation. Visual inspection confirmed the complete solubilisation (3D collagen scaffolds turned white from dark violet). Absorbance was measured at 550 nm and viability was calculated as the percentage of absorbance of treated cells relative to untreated controls for each time point (Figure S4). Experiments were conducted twice, with three replicates each time.

### Flow cytometry

For cell cycle analysis (Figure S5), cells were harvested by trypsinization, and the resulting pellets were fixed in cold 70% ethanol with gentle agitation and stored at − 20 °C overnight. The cell suspension was stained with a solution containing propidium iodide (PI; 200 µg/mL), RNAse (20 mg/mL), and NP40 (0.15%) for 24 h at 4 °C. The samples were then analyzed the following day using an Attune NxT Flow Cytometer (Invitrogen), and the data were processed with FlowJo software. Each sample was run in duplicate, with 10,000 events recorded per replicate.

### FO-LTDR in vitro testing on breast cancer line

The in vitro drug release assays on cancer cell lines required the design and development of a suitable microfluidic chip to control the movement of fluids through micrometer-sized channels. The final 3D-printed resin device (manufactured by SanChip S.r.l, Italy) consisted of a 4 cm long main channel with an inner diameter of 3 mm for the fiber platform housing and a secondary channel inclined at 45 degrees as an inlet for the buffer solution to ensure constant hydration of the polymeric carriers deposited over the fiber. A 1 mm × 1 mm pillar system in the center of the main channel was designed to ensure both the robustness of the structure and to minimize heat dissipation, as it represents the only connection point between the main channel and the outer part of the chip. The outlet has been fitted with a matching conical medical needle adapter for the drug release collection. A peristaltic pump (Watson-Marlow—403U/VM2 10 RPM -) was used to transfer 100 µl of buffer solution from a silicone tube into the secondary channel of the microfluidic device until the fluid reached the main channel. During the experimental tests, a suitable cap with a central hole of 250 μm diameter was attached to prevent leakage of the liquid, and the fiber probe that fit through this cap was connected to the same experimental setup as previously described. The laser pumping system was then activated (input power = 450 mW) and then the value of the injected power was slightly modified to ensure a constant temperature above 50 °C on the metallic layer.

The optical activation of the FO-LTDR device was performed inside the microfluidic platform used for drug release experiments. The DOX released form the platform was collected during the activation time in the flowing buffer solution and subsequently transferred to the cell cultures for biological evaluation. Within the following setting the MCF7 cells were not directly exposed to the optical fiber or to the localized heating generated during laser activation.

Under these conditions, MCF7 cell viability following FO-LTDR activation was evaluated (as described in the previous paragraph) and the observed cytotoxic effect can therefore be attributed exclusively to the released drug rather than to thermal stimulation.

### Statistical analysis

All the experiments were performed at least three times by means of identical devices tested under the same experimental conditions. Statistics are expressed as mean ± relative standard deviation that was determined by dividing the standard deviation by the mean value. Statistical significance was determined using a two-tailed Student’s t-test, with p-values < 0.05 considered significant.

## Results

### Microgel synthesis and characterization

**Fig. 1:**
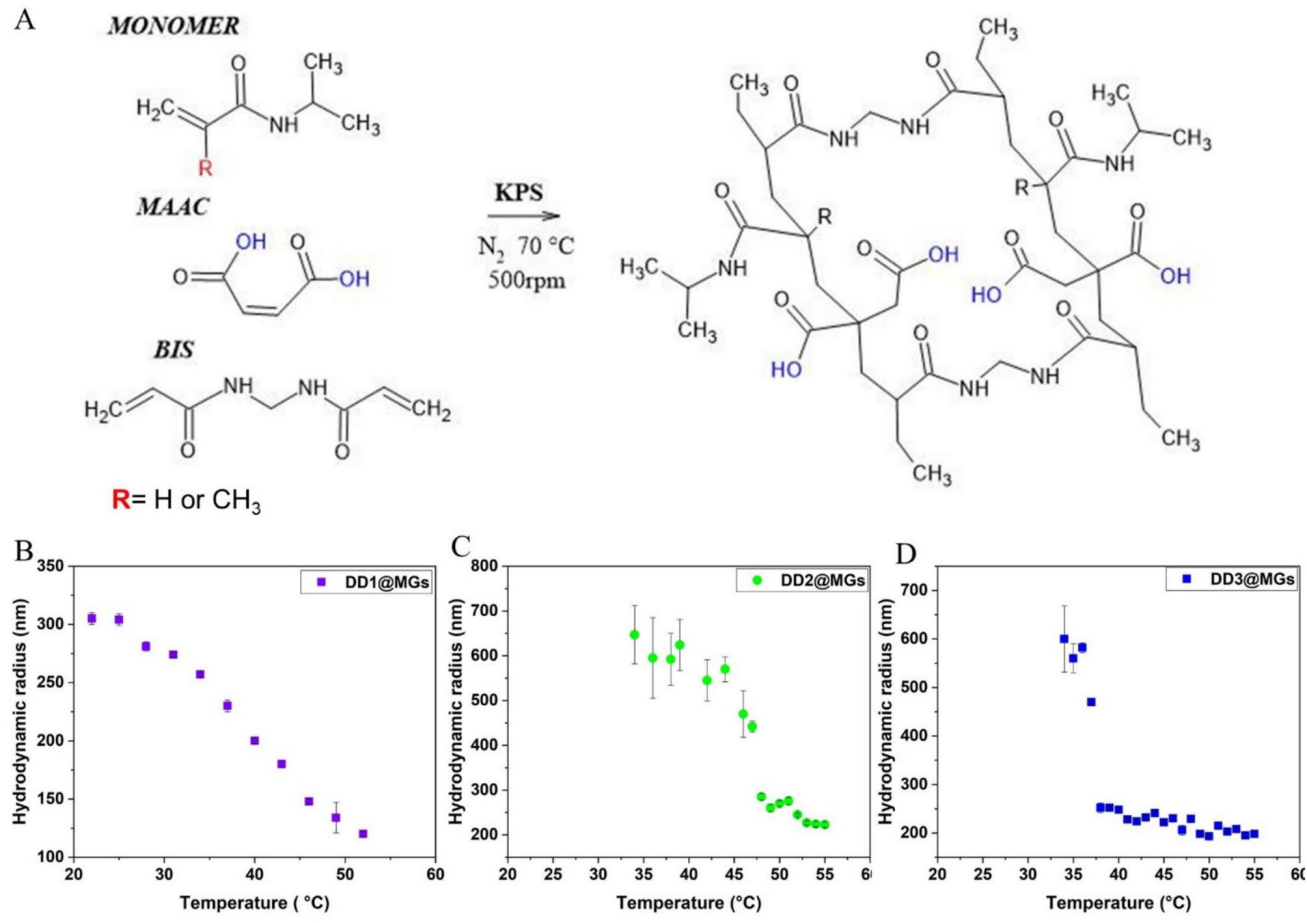
Microgels are synthesized via free radical polymerization (**A**) using thermoresponsive monomers (Nipam R = H/Nipmam R = CH_3_), methylenebis-acrylamide (BIS) as a cross-linker, and maleic acid (MAAC). Dynamic light scattering (DLS) analysis of the different formulations—DD1 (**B**), DD2 (**C**), and DD3 (**D**)—reveals their distinct volume phase transitions.

MGs with different monomer ratios, crosslinker and maleic acid amounts were synthesized by free radical polymerization reaction. A few minutes after the addition of the initiator KPS, anionic sulphate radicals (SO_4_^−^) initiated the copolymerization of NIPAM/NIPMAM and the MAAC simultaneously with BIS as a cross-linker (Fig. 1A). As an indication of MGs formation, a milky white solution was observed. Both NIPAM and NIPMAM were used to obtain thermo-responsive MGs labelled DD1, DD2 and DD3, which undergo a volume phase temperature transition (VPTT) in 37–45 °C range.

The thermo-responsivity of MGs was investigated by DLS. As shown in Fig. 1C, D, for the MGs DD2 and DD3, it was not possible to measure the hydrodynamic radius with good sensitivity under 34 °C, because the MGs swell and the scattering resulted in too weak a signal to be detected. The hydrodynamic radius of the MGs-DD2 and DD3 was about 600 nm at 34 °C, while the radius of DD1 was about half of that in the same condition of analysis.

The degree of thermally-induced shrinkage for DD2 and DD3 was measured to be 69.86% and 56.81%, respectively, between 37 and 52 °C. In contrast, DD1 exhibited a shrinkage of 24.3% between 25 and 37 °C and shrank by a further 47.8% in the range 37–50 °C. As can be seen in Fig. 1C, the VPTT for DD2 is above 42 °C, while DD1 and DD3 have VPTTs close to the physiological temperature of approximately 37 °C (Fig. 1B–D). The upward shift of DD2 can be attributed to the presence of NIPMAM in its synthesis, which results in a higher transition temperature due to an additional methyl group compared to NIPAM. Among the formulations, DD2 was selected for further investigation as it exhibited improved shrinking capability and a shifted VPTT. These properties enable more precise control of DOX release, activated by external thermal triggers, thereby preventing the shrinkage of the MGs under physiological conditions (37 °C).

**Fig. 2:**
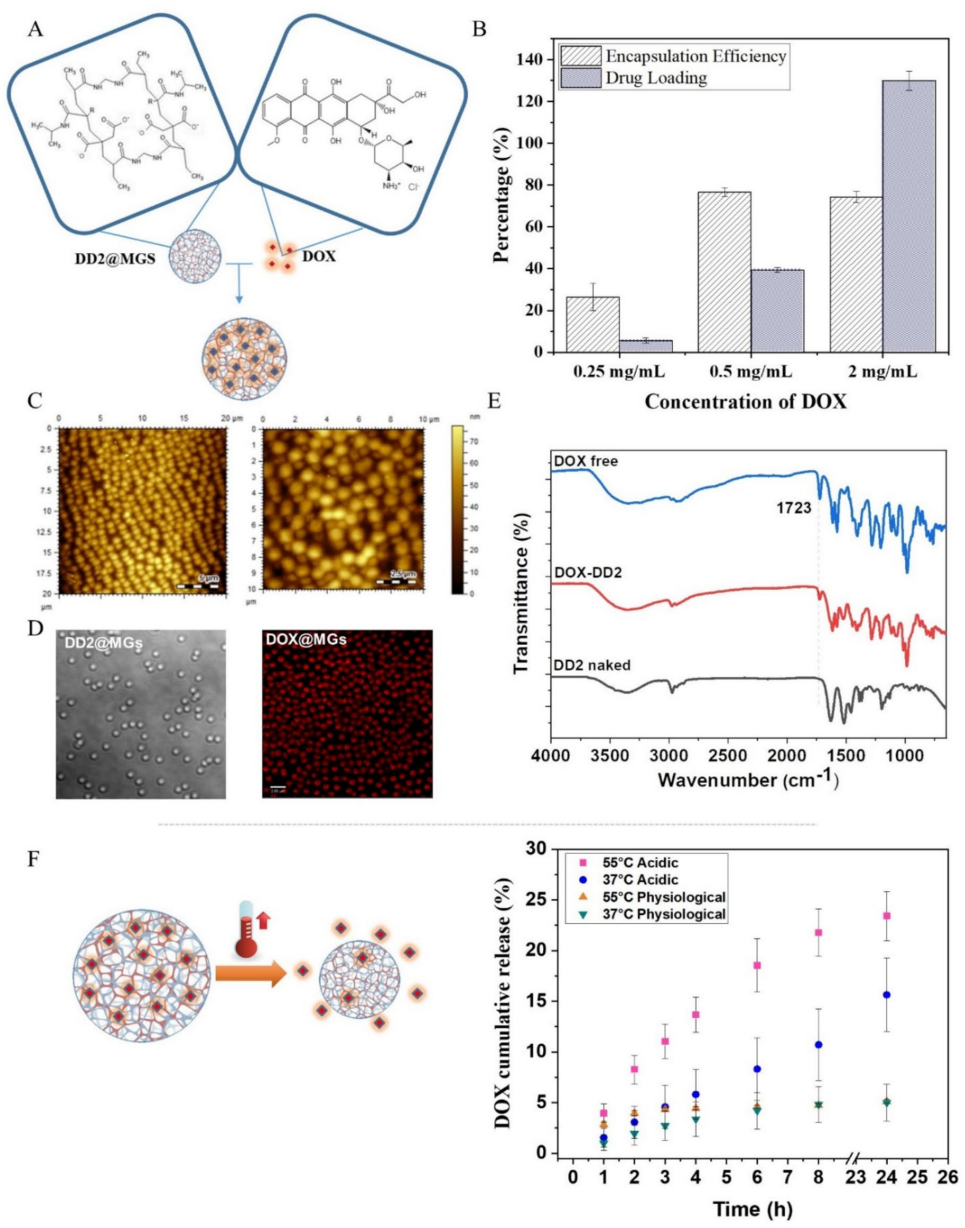
(**A**) Doxorubicin (DOX) is incorporated into the polymer network through electrostatic interactions between the protonated amino groups of the drug and the deprotonated carboxylic groups of MAAC under loading conditions (PBS, pH 7.4). MGs characterization: (**B**) Encapsulation efficiency (EE) and drug loading (DL) of DD2 MGs expressed in percentage (%); (**C**) AFM images at different magnification (scale bar 5 um and 2.5 um) of dried MGs deposited on gold planar substrate; (**D**) CLSM images of MGs: on the left, MGs acquired in Bright-field mode (objective 63 × 1.1 oil; on the right fluorescence emission of MGs after the loading procedure (excitation 484 nm, emission from 496 to 600 nm—scale bar 2 um; (**E**) FTIR spectra of empty and loaded MGs compared with free DOX (black, red and blue line respectively). (**F**) Scheme of DOX release mechanism triggered by the external stimulus: Drug release profiles of DOX@MGs measured at different Temperature and buffers.

When freeze-dried DD2 are immersed in an aqueous DOX solution at pH 7.4, they swell and entrap the drug within their polymer network. This loading process, which takes place at physiological pH, relies on the electrostatic interaction between the positively charged DOX molecules and the negatively charged carboxylic groups of the maleic acid units in DD2 (Fig. 2A). To prevent undesirable precipitation at this pH, DOX was used in its hydrochloride form. The EE and DL can be tuned by varying the concentration of DOX used for the loading, as shown in Fig. 2B. In particular, 1 mg of DD2 incubated with 0.25, 0.5 and 2 mg/mL of DOX achieved DL of 5.6 ± 1.4%, 34.4 ± 1.1%, 130 ± 4.6% and EE of 26.4 ± 6.6%, 76.6 ± 2.1%, 74.3 ± 2.6% (see section “Introduction” in the supplementary material). The drug concentrations of 0.25, 0.5, and 2 mg mL⁻^1^ were selected to explore different loading regimes within the thermoresponsive microgel system. The lowest concentration (0.25 mg mL⁻^1^) was used to evaluate the baseline drug loading under limited drug availability, whereas 0.5 mg mL⁻^1^ represents an intermediate condition allowing the progressive increase of drug uptake to be assessed. The highest concentration (2 mg mL⁻^1^) was chosen to approach the upper loading capacity of the microgels network. Based on the loading results, the formulation loaded with 2 mg/mL of DOX, referred to as DOX@MGs, was selected for all further studies.

We also performed DOX@MGs characterizations through AFM, CLSM, and FTIR Spectroscopy analysis. AFM analysis demonstrated that DOX@MGs possess a regular shape and uniform dimensions (Fig. 2C), with no observable morphological defects or irregularities. CLSM images validate the AFM findings, demonstrating that MGs are monodisperse, homogeneous, and possess a spherical morphology (Fig. 2D, left). Additionally, the intense fluorescence observed in Fig. 2D (right) after the loading procedure confirms the successful encapsulation of DOX within the MGs. FTIR spectrum of DOX (Fig. 2E) shows the characteristic peaks at 1723 cm^−1^ related to carbonyl groups of cyclohexanone ring, 1575 cm^−1^ and 1616 cm^−1^ belonged to the aromatic C = C stretch. These peaks are also observed in DOX@MGs, confirming drug loading. Not loaded MGs (DD2 naked) and DOX@MGs share a distinctive peak at: 1633 cm^−1^ due to the stretching vibration of the C = O group; 1527 cm^−1^ due to the bending vibration of the amide groups, and the peaks at 2976 and 2838 cm^−1^ caused by the asymmetric and symmetric stretching vibrations of the C–H bond from methyl groups. The broad peak around 3346 cm^−1^ is due to the N–H stretching vibration of the amide. Finally, the release profile of DOX was then investigated under different pH and temperature conditions. As shown in Fig. 2F, approximately 23.4% (0.3 mg/mL) of DOX was released at 55 °C in an acidic buffer after 8 h. By comparison, only 15.6% (0.15 mg/mL) was released in the same buffer at 37 °C, confirming a temperature-dependent release pattern.

Almost all previously published studies on Dox-loaded thermoresponsive MGs are mainly based on NIPAM (Table 1), often in combination with ionic comonomers (either anionic, such as acrylic acid, or cationic, such as (dimethyl)aminopropylmethacrylamide) together with a crosslinker. Consistent with these already proposed NIPAM-based formulations (Table 1), our DOX@MGs achieved an EE of 74.3%, which is within the reported range of 63.6–90%. Notably, our formulation also demonstrated a markedly higher DL of 130%, which is significantly higher than the typical range of 5–20%. This can be attributed to the inclusion of maleic acid as an anionic component. In support of this, Dhanya et al.^57^ achieved a comparable DL (127.2%) in NIPAM-maleic acid MGs, although with a lower EE of 63.6% (Table 1). Regarding the T-phase transition, while DOX-loaded MGs in Table 1 typically exhibit transitions between 37 and 42 °C (close to physiological temperature), our formulation showed a higher volume phase transition temperature. This shift could help prevent the non-specific release of DOX under physiological conditions, thereby enhancing its potential for in vivo applications as a thermoresponsive drug delivery system triggered by exogenous stimulus.

**Table 1:** Comparison between different preparations of MGs for DOX release.

	Monomers	Encapsulation efficiency (EE %)	Drug loading (DL %)	Release in physiological buffer	Release in acidic buffer
Our	NIPMAM MA	74.3 ± 2.6	130 ± 4.6	8 h 3% 37C 8 h 3.5% 55C	8 h 10.7% 37C 8 h 21.4% 55C
^61^	Fe_3_O_4_ NIPAAM-DMAEMA MBA	75	6.9	400 h 25% 37C	400 h 60% 37°c 400 h 70% 40 °C
^62^	NIPAM-co-5%AA nanogel embedded in hydrogel	90	19	24 h 50% 37C	24 h 90% 37C
^63^	NIPAM; AA	92.7	18.5	8 h 7% 25C 8 h 10% 37C	8 h 18% 25C 8 h 24% 37C
^64^	AA; EGDMA	–	22	8 h 20%–10% 37C	8 h 30%–15% 37C
^57^	NIPAM; MA;MBA	63.6	127.2	–	4 h 10% 37C 4 h 46% 41C
^65^	NIPAM; AA; MBA	–	–	8 h 45% 37C 48 h 85% 37C	–
^66^	NIPAM; MAA; MBA	–	–	10 min 65% 37C	–
^67^	NIPAM; PEI	80.6 ± 4.5	72.8 ± 2.3	72 h 20% 37C 72 h 40% 42C	72 h 40% 37C 72 h 70% 42C
^68^	NIPAM; BM; BIS	–	–	7d 6% 37C	7d 28.2% 37C
^69^	NIPAM; AAm; BIS	–	4.9	96 h 38% 37C 96 h 76% 42C	96 h 46% 37C 96 h 96% 42C

Encapsulation Efficiency (EE) measures the percentage of the initial amount of drug that has been successfully trapped or encapsulated within a carrier system. Drug Loading (DL) measures the weight ratio of the drug to the total weight of the carrier.

DMAEMA = N,N-dimethyl-aminoethyl methacrylate; NIPAM = N-isopropylacrylamide AA = acrilyc acid;; MA = maleic acid;MAA = methacrylic acid; MBA = N,N′-methylenebis(acrylamide); NIPMAM = N-isopropylmethacrylamide, EGDMA = ethylene glycol dimethacrylate; PEI = polyethylenimine; BM = butyl methylacrylate; AAm = acrylamide

In terms of release kinetics, the chemistry of our NIPMAM-based formulation effectively minimizes the release of DOX under physiological conditions. In fact, less than 5% of DOX is released in our formulation at physiological pH and 37 °C, while about 10% was reported for previously published NIPAM-maleic acid MGs^57^. This improved stability is attributed to stronger electrostatic interactions at physiological pH, which allow the polymer network to retain the drug. Under acidic conditions, where electrostatic interactions are weakened, and at temperatures above the T-phase transition, our NIPMAM-maleic acid formulation showed a more sustained release compared to the pronounced DOX release of 46% within 4 h at 41 °C observed in the aforementioned study^57^. This difference in release kinetics could likely be related to the architecture of the polymer network: as noted by Wedel et al.^60^, NIPAM MGs have a tightly cross-linked core and a looser cross-linked shell that allows for faster drug release. In contrast, the homogeneously cross-linked structure of NIPMAM MGs supports a slower, sustained release.

### Fiber heating platform and light-mediated drug release

Previous studies were conducted to determine the optimal fabrication parameters of the fiber heating platform in terms of core-offset dimension, Au-layer thickness and length, with the aim of improving the light coupling into the cladding and, consequently, to enhance the heating efficiency of the platform^50^. The FOH device was entirely implemented using a standard single-mode optical fiber (acrylate SMF28e) by creating a 6 µm core-offset junction (*Figure S1*), which enabled 90% of the light propagating inside the core to be coupled into the cladding. The core-offset was fabricated 2.5 mm away from the 150 nm-thick gold-coated section of the fiber, inside which a 5 mm-long commercial FBG was integrated. The Au thickness was fixed at 150 nm since which was demonstreded in previous study to ensure the complete extinction of the cladding mode electric field before it reaches the external medium, while maintaining a reduced thermal mass of the system^50^. A further increase in the coating thickness and/or length would result in an undesired increase of the thermal mass, negatively affecting the heating efficiency of the device.

To demonstrate the feasibility of tailoring the proposed platform to specific payload requirements, two different Au coating lengths (*l* = 10 mm and 20 mm) were investigated. Since the amount of immobilized microgels—and therefore the total releasable drug—scales with the available coated surface area, adjusting the length of the Au-coated region provides a straightforward strategy to modulate the drug loading capacity of the FO-LTDR platform. The FOH performance was investigated in terms of thermal gradient generated per unit input power, by evaluating the temperature rise in the gold-coated region at incremental values of the injected power.

**Fig. 3:**
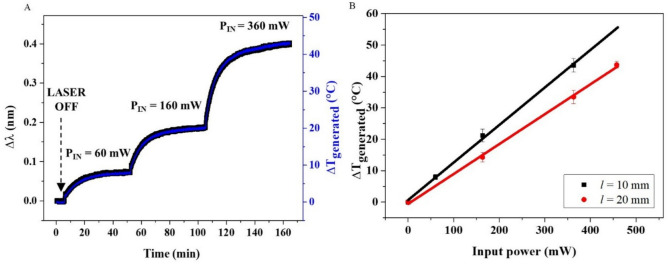
(**A**) Typical response of the proposed FOH in water at incremental injected power values for a device characterized by l = 10 mm. (**B**) Thermal gradient vs light input power calibration curves for the two devices characterized by l = 10 and l = 20 mm. The heating efficiencies are calculated as the slope of these curves.

Figure 3A shows the typical response of a device with *l* = 10 mm at incremental injected power values up to ~ 360 mW, in water. As expected, a red shift of the Bragg wavelength was recorded at each power injection (black curve), corresponding to a gradual increase in the temperature of the Au layer (blue curve), which was retrieved by considering the (previously measured) FBG sensitivity of 9.3 pm/°C. Overall, a temperature variation of 43 °C was registered in correspondence with the maximum applied power value. The heating efficiency, as measured by the slope of the linear curves fitting the ΔT_generated_–Input power curves in Fig. 3B, was determined to be ~ 12.2 °C/100 mW (R^2^ = 0.99) and ~ 9.4 °C/100 mW (R^2^ = 0.99) for the devices with *l* = 10 mm and *l* = 20 mm, respectively. As expected, the shorter the Au layer, the lower the thermal mass and, consequently, the higher the heating efficiency. Anyhow, both values of the efficiency are slightly better than the one reported by Zhang et al.^46^ and can be further enhanced by judiciously transforming the gold layer in a plasmonic resonator, allowing for ultra-high light absorption phenomena to improve energy conversion efficiency.

The FOH platforms were successively integrated with DOX@MGs yielding the fully assembled drug delivery system. The integration was performed along the lateral surface of the fiber using a dip coating approach, and their integration was verified by CLSM (*Figure S2*). As shown in *Figure S2*, the optimized deposition protocol enables the immobilization of a monodisperse and homogeneous layer of DOX@MGs on the gold surface of the FOH. The MGs coverage obtained along the surface clearly demonstrates the lack of multilayers, which could affect the controlled release, while the covalent immobilization guarantees the required stability for LTDR application.

For each fiber platform configuration (i.e., *l* = 10 mm and *l* = 20 mm), the device was “internally activated” by launching light with a sufficient input power. This power induced a surface temperature increase, reaching the VPTT of the MGs (Figure S3). This process, in turn, caused the MG collapse, thereby triggering the DOX release. The irradiation duration used in this study was selected to ensure that the temperature of the Au-coated region remained above the VPTT of the microgels for a sufficiently long period to fully characterize the release profile and quantify the maximum drug payload delivered by the FO-LTDR platform.

**Fig. 4:**
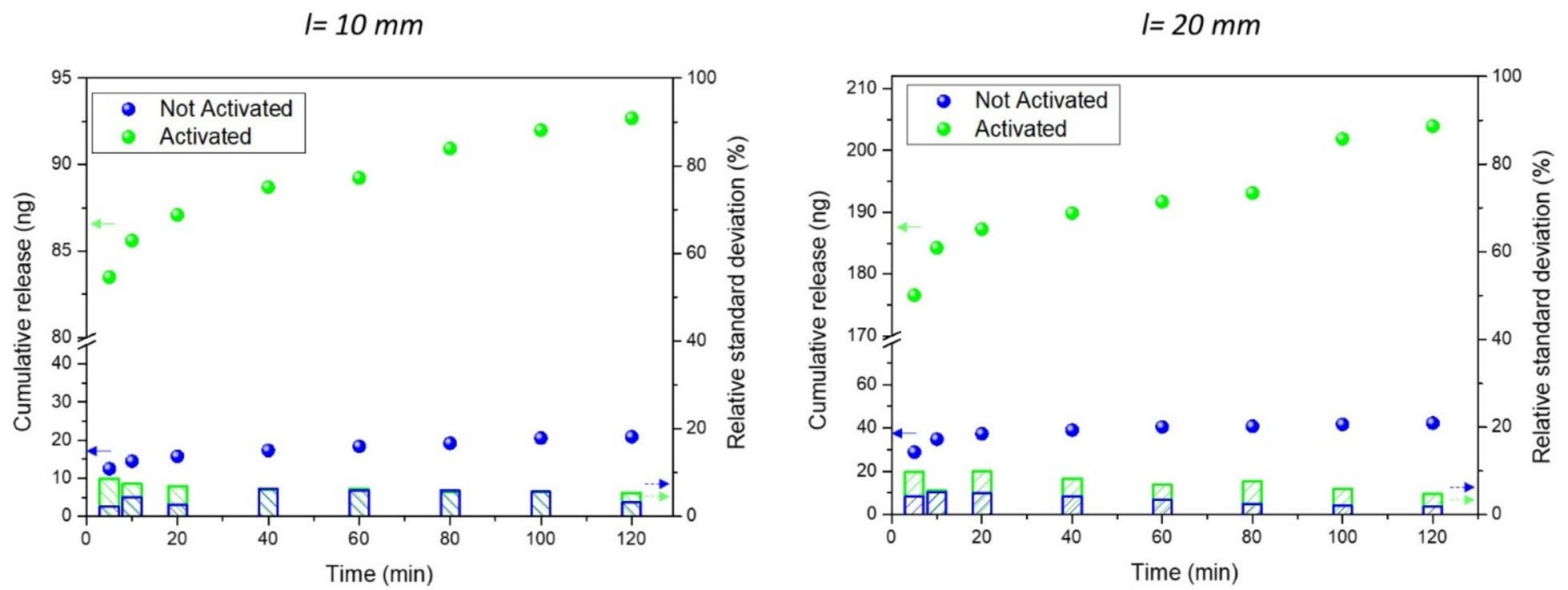
In vitro cumulative drug release performed by the proposed platform characterized by (**A**) 10 mm-long and (**B**) 20 mm long Au coating.

The cumulative release was then compared to an identical “*non-activated*” probe (i.e., a probe inside which no light was launched). Figure 4 shows the cumulative DOX release from fiber optic platforms with Au-coated regions of 10 mm (A) and 20 mm (B). For the 10 mm device, most DOX (~ 78 ng) was released within the first 5 min upon internal activation, with an additional ~ 10.6 ng released gradually, reaching a total of ~ 89 ng (green circles), compared to ~ 20 ng in non-activated controls (blue circles). Similarly, the 20 mm fiber released ~ 176 ng in the first 5 min and an additional ~ 28 ng over the next 110 min, reflecting the higher payload due to the longer gold-coated region.

The initial burst release observed during the first ~ 20 min is consistent with the rapid thermally induced collapse of the microgel network once the temperature exceeds the VPTT.

Both configurations exhibited high reproducibility, with relative uncertainties below 10%, thereby highlighting the reliability of the proposed platform and its potential as a fast, controllable, and scalable solution for drug delivery applications. Overall, the significant gap between the total amount of DOX that the platform (20 mm) was able to release with the light activation (~ 204 ng), combined with the negligible rate of DOX leakage (~ 40 ng during the first five minutes of the negative control tests), indicate the effectiveness of the proposed platform and pave the way for its potential exploitation for loco-regional light-triggered drug release. Owing to the optical nature of the activation mechanism, the FO-LTDR platform inherently enables temporal control of drug release. In principle, different activation strategies—including shorter irradiation periods, pulsed laser operation, or repeated activation cycles—could allow ON–OFF switching of drug release and tunable therapeutic time windows.Achieved results also demonstrated the possibility of using the length and diameter of the Au-coated region as a degree of freedom for the proposed platform to match the payload requirements of various medical applications.

### Biological activity of DOX on breast cancer cell line MCF7 in 2D and 3D culture model

To assess the cytotoxic effect of DOX, the breast cancer cell line MCF7 has been identified as the experimental model. MCF7 is a human breast cancer cell line derived from luminal A histotype positive for estrogen receptor (ER+). The in vitro efficacy of free DOX was investigated after 72 h in MCF7 cells cultured either in a standard monolayer culture or in a 3D culture with collagen-based scaffolds.

In order to determine the optimal DOX concentration to be released by the optical fiber probe, pharmacokinetic studies in breast cancer patients treated with anthracyclines were considered, identifying the maximum concentration of DOX and its metabolites at 1.6 µg/mL after two cycles of bolus therapy at 50 mg/m^2^^70^. According to the guidelines of the Italian Association of Medical Oncology (AIOM), the recommended dose for breast cancer treatment is between 60 and 75 mg/m^2^, either as monotherapy or in combination (with a cumulative dose not exceeding 550 mg/m^2^). Based on this, a conversion was made from the peak plasma concentration obtained from the pharmacokinetic studies referenced to the concentrations currently used in clinical practice, resulting in an optimal concentration of 2.4 µg/mL. However, considering the translational application of our study, which consists of locoregional therapy—with minimal concentration of drug locally administered—a lower range of concentrations was also tested in both 2D standard monolayer, 6-well plates and 3D cultures.

As shown in Figure S4, after 72 h the plasma peak concentration of free DOX (2.4 µg/mL) resulted in an inhibition of cell proliferation of about 70% in MCF7 2D cultures, while in 3D cultures inhibition of cell proliferation reached 30%. Decreasing the concentration from 0.6 to 1.2 µg/mL, we observed an inhibition of proliferation comparable to the value obtained at plasma peak, while in 3D cultures inhibition of cell proliferation was about 15%. Moreover, in the 3D scaffold model, 72 h exposure to free DOX did not result in greater inhibition of MCF7 cell proliferation at 0.6 and 1.2 µg/mL compared with 0.15 and 0.3 µg/mL. This plateau in cytotoxic efficacy, consistent with previous reports^71,72^, reflects the non-linear dose–response typical of 3D systems, where drug penetration barriers, reduced proliferative activity in hypoxic core regions (doxorubicin is more effective against proliferating cells), and activation of matrix- and stress-induced resistance mechanisms limit the benefit of higher nominal concentrations.

Furthermore, as expected, the survival percentages on the scaffolds (3D) are significantly higher compared to those in monolayer culture (2D) at all tested concentrations (*Figure S4* dashed line). This trend aligns with our previously published data^71,72^, which demonstrated increased drug resistance in cells cultured in 3D compared to 2D. Since the 3D culture model most closely mimics in vivo conditions, the observed findings indicate that the lower drug dose is adequate to effectively inhibit cell proliferation.

These initial optimization steps suggest 1.2 μg/mL as the optimal effective concentration. As a complementary investigation, a cell cycle analysis was performed after DOX treatment (1.2 μg/mL) at different time points on MCF7 cell lines. As shown in Table 2 and *Figure S5*, at all-time points, a reduction in the percentage of cells in the G0/G1 phase and an increase in the percentage in the S phase were observed compared to the control. This suggests that the treatment induces a block in the S phase, preventing progression past the G2 checkpoint. Thus, based on these findings, we selected 1.2 μg/mL as the optimal effective concentration for FO-LTDR treatment.

**Table 2:** Cell cycle analysis on MCF7 cell line not treated (CTRL) and treated with free DOX after 24, 48 and 72 h. Values represents percentage of cell in each phase.

		G0/G1 (%)	S (%)	G2/M (%)
24 h	CTRL	45.40	32.46	22.14
	free DOX	27.84	48.64	23. 52
48 h	CTRL	59.58	29.95	10.47
	free DOX	26.90	48.66	24.44
72 h	CTRL	63.02	26.82	10.16
	free DOX	26.77	42.26	30.94

### In vitro light-to-heat triggered drug release

The selection of the most suitable FO-LTDR platform was guided by the requirements identified through both the biological analysis and the specifications of the locoregional delivery approach.

**Fig. 5:**
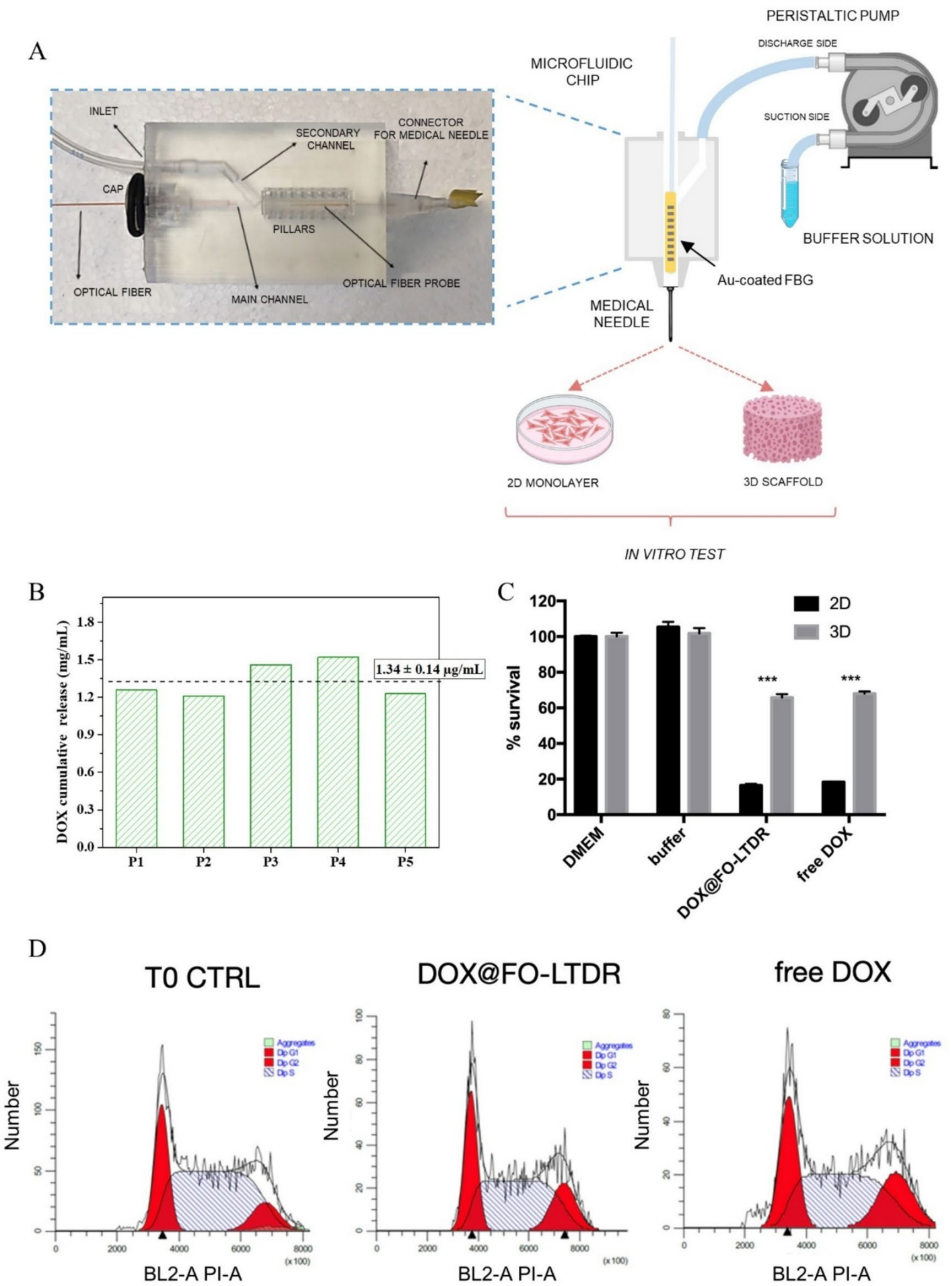
(**A**) Experimental set-up used for in vitro drug release tests on the cancer cell lines. (**B**) Quantification of DOX release from five different FO-LTDR probes in microfluidic chip. (**C**) Evaluation of the in vitro MCF7 cell viability upon the treatment with free DOX or DOX directly released from FO-LTDR in 2D and 3D after 72 h. (**D**) Cell cycle analysis of the MCF7 cell line untreated (CTRL left graph), or treated with the DOX released from FO-LTDR (middle graph) or free DOX (right graph) after 72 h.

In particular, the DOX dose required for treatment, along with the constraint of delivering the drug within a limited solution volume (≤ 100 mL), made the platform with a length of 20 mm especially suitable for the intended in vitro application on the breast cancer cell line. For this purpose, a microfluidic device (Fig. 5A) was designed to precisely integrate the optical probe. The fiber device was inserted into a specially modified channel (designed to minimize thermal losses) through an inlet that ensured the tightness of the fiber and prevented fluid leakage. A buffer solution was perfused through a second inlet at a flow rate of 50 µL/min using a peristaltic pump. The final microfluidic system allows the control of the DOX release and its delivery to the site of interest thanks to the modified outlet specifically adapted for attaching a needle. Experimental results from in vitro release tests conducted on the chip, using five probes from the same fabrication batch (Fig. 5B), confirmed a consistent and reliable DOX release, yielding an average concentration of 1.34 ± 0.14 µg/mL. Following the FO-LTDR activation the solution contained the drug released in the chip was conveyed to cell cultures both in 2D and 3D.

As shown in Fig. 5C, in 2D cultures the DOX released after the FO-LTDR activation showed a nearly identical survival rate compared to the free DOX at equal concentration (20%), with excellent reproducibility between different tests (standard deviation ± 0.94 in 2D and ± 1.89 in 3D settings). As expected, in 3D cultures the drug showed reduced efficacy compared to 2D cultures, both when administered in its free form and when delivered through the optical fiber probe. Specifically, in the MCF7 line, a nearly identical survival percentage is observed between DOX released from the probe and free DOX in both 2D (20%) and 3D (60%), with excellent reproducibility. These findings prove that the FO-LTDR platform enables the delivery of drug to the tumor cells without compromising the efficacy of the “passenger drug”. Furthermore, the cell cycle analysis was performed after the release of the DOX induced by the shrinking of the DOX@MGs bound on the FOH probe. As shown in Fig. 5D, 72 h after the treatment, the cell lines exhibited a reduction in the percentage of cells in the G0/G1 phase and an increase in the percentage in the S phase compared to the control, confirming that the treatment causes a block in the S phase, preventing progression to the G2 checkpoint. The data confirm that DOX released from FO-LTDR, retains the same cell proliferation inhibition activity.

These findings are highly significant, as they demonstrate that our platform can deliver therapeutically relevant drug concentrations at the target site while avoiding higher systemic doses and reducing the risk of side effects.

Concerning the safety of the platform, while the covalent bond of the microgels guarantees the selective release of the drug, even in the unlikely event of material leakage, cytotoxic effects can be excluded, as biocompatibility assays on DD2 (not loaded MGs) have confirmed their intrinsic non-toxicity (data not shown).

## Discussion

In this work we propose a novel nanophotonic platform, based on the integration of an all-fiber heater device with thermo-responsive MGs@DOX, with the potential to provide targeted, light-triggered release of DOX through a light-to-heat conversion mechanism (with thermal heating efficiency up to ~ 12.2 °C/100 mW). This approach leverages the MGs ability to respond to local environmental changes, ensuring precise, controlled drug release directly at the tumor site. The platform’s simple fabrication and adaptable design enable local delivery of therapeutic DOX levels, maximizing efficacy while minimizing systemic exposure. The therapeutic performance of the fiber platform was validated in breast cancer models. Effective drug delivery was demonstrated in both conventional 2D cell cultures and advanced 3D scaffolds that closely mimic the structure and behavior of the tumor tissue. The use of 3D scaffolds enables a more accurate representation of in vivo tumor conditions, underscoring the platform’s potential to improve drug delivery and therapeutic outcomes in complex tissue environments. Although optical fibers have been extensively investigated by scientific communities for PTT and PDT, the concept of using optical fiber-based platforms as light-triggered drug delivery systems represents a new paradigm in the therapeutic scenario, with only a few studies addressing this concept^44–46^. One of the pioneering examples was demonstrated by Nazari et al.^44^, who designed an optical fiber coated with a metal–organic framework (MOF), specifically UiO-66, to deliver the anticancer drug 5-FU. The drug was loaded into the MOF’s porous structure and subsequently released through a fiber-triggered sublimation process. While this minimally invasive approach showed promising potential for drug delivery, it required a substantial number of fibers (between 111 and 185) to achieve therapeutic cytotoxicity, underscoring a significant limitation in scalability. More recently, Zhang et al. developed a system for treating hepatocellular carcinoma, where an optical fiber induced localized heating, leading to the release of a drug encapsulated within melted agarose^46^. The heat generated by the fiber facilitated the release of both free drug and agarose microstructures containing the drug directly into the tumor site. While this approach demonstrated the efficacy of local drug delivery, it also highlighted the challenges associated with using agarose as a triggered delivery material. Agarose has a relatively high upper critical solution temperature (UCST) and a slow degradation rate, which can cause prolonged presence and potential accumulation in tissues, raising concerns over drug clearance. Furthermore, despite its general biocompatibility, agarose can elicit immune responses, particularly in long-term applications, which could limit its feasibility for clinical use. Conversely, the nanophotonic platform proposed here addresses the limitations identified in previous studies by enhancing chemotherapy delivery with a focus on advancing personalized medicine. This platform enables the precise, targeted release of pure drugs directly at the site of interest, triggered by the light-based thermo-activation. The key to this process is the covalent coupling of DOX-loaded MGs onto the lateral surface of the optical fiber platform. This strategy facilitates the homogeneous distribution of drug-loaded MGs over the entire active probe surface, thereby promoting consistent and precisely regulated drug release. The covalent binding of DOX@MGs offers a significant improvement as it prevents the inadvertent leakage of other materials or excipients that could alter the drug’s pharmacokinetics. In addition, the proposed platform enables the fine-tuning of drug release via a light-induced thermal stimulus, which triggers the controlled collapse of the immobilized DOX@MGs. This feature enables precise modulation of release, increasing flexibility and efficiency without the need for customized fiber settings for different drug loading capacities. In addition, compared to the device proposed in^46^—that is fabricated by cascading a multimode/rare-earth-doped single-mode (SM)/multimode optical fiber structure, followed by a complex and manually operated integration of a mm thick film of agarose and doxorubicin—the proposed platform is easier to fabricate and much more prone to multiplexed manufacturing processes, therefore allowing for huge time and costs savings. Finally, it provides the possibility to capitalize on several degrees of freedom to suit the required payload for the specific medical applications. To the best of our knowledge, our thermoresponsive NIPMAM–maleic acid-based MGs are the first to be coupled with an optical fiber for light-induced drug delivery. Their tailored properties enable the stable retention of DOX within the polymer under physiological conditions at 37 °C, while allowing the locoregional release of the drug upon specific light stimulus.

Overall, the proposed platform offers a straightforward, cost-effective, and efficient strategy for site-specific chemotherapy, characterized by consistent performance and adaptability, thereby contributing to a refined and personalized therapeutic approach. Therefore, when the FO-LTDR is tested in vitro, we can locally trigger the release of DOX in breast cancer cells, inducing a cytotoxic effect. In particular, the cytotoxicity data showed that DOX released via FO-LTDR had similar efficacy to that of free DOX, with low variability between different probes. This indicates that the FO-LTDR system can successfully deliver DOX to target cells, suggesting its potential application in locoregional chemotherapy for solid tumors.

The proposed FO-LTDR platform offers broad translational potential across various oncological contexts, enabling integration into existing treatment modalities. Given the potential applications that make this device highly attractive for oncological interventions, particular emphasis should be placed on its straightforward adaptability to a broad spectrum of malignancies amenable to locoregional therapeutic approaches. In such contexts, reduced yet tumor-focused doses of chemotherapeutic agents can be selectively delivered to anatomically challenging sites, thereby minimizing systemic exposure and toxicity while preserving robust therapeutic efficacy at the tumor site. For ovarian cancer, it could enhance intraperitoneal chemotherapy for disseminated tumors, complementing hyperthermic approaches. In colorectal cancer with liver metastases, it facilitates minimally invasive, targeted drug delivery to metastatic lesions. Its precision also could address site-specific challenges in soft tissue sarcomas, effectively treating recurrent or unresectable tumors with minimal systemic exposure. Furthermore, in the case of esophageal cancer, the platform’s compatibility with endoscopic procedures could support controlled drug release for improved outcomes in advanced or resistant cases. Notably, the FO-LTDR platform can be also upgraded to deliver PDT or PTT therapies, thus enabling a synergistic treatment modality that augments antitumor efficacy and improves therapeutic outcomes. In this context, the inherent versatility of optical fiber–based systems also offer the possibility of integrating multiple independently addressable FO-LTDR units within the same catheter or fiber bundle, potentially enabling spatially controlled drug release across different tumor regions through the selective activation of individual fibers. Such an approach could allow the generation of drug concentration gradients or multi-site therapeutic interventions in heterogeneous tumor environments. Furthermore, the integration of advanced sensing functionalities—such as drug release monitoring via the inscription of a long-period grating within the core of the MGs-coated fiber^73–75^, or therapy monitoring through the implementation of metasurface-enhanced Lab-on-Fiber biosensors at the fiber tip^76–80^ would represent a significant step toward the development of a fully integrated, all-in-one theranostic platform.

## Conclusion

Conventional systemic chemotherapy faces challenges such as off-target toxicity, limited specificity and the inability to effectively combat treatment resistance. To overcome these limitations, locoregional drug delivery platforms offer promising solutions that enable precise, site-specific delivery of therapeutics while minimizing systemic exposure. In this work, we have presented a novel nanophotonic platform that combines the advantages of fiber optic technology with the functional versatility of thermoresponsive microgels. The FO-LTDR platform enables the local, light-controlled doxorubicin release by utilizing the efficient light-to-heat conversion of a fiber optic heater with covalently immobilized DOX@MGs. The thermal responsiveness of the MGs allows for a highly tunable and controlled release mechanism that is activated by precise external stimuli. Validation in 2D and advanced 3D breast cancer models demonstrated the platform’s robust efficacy, closely mimicking in vivo conditions and underscoring its potential for precise, site-specific chemotherapy.

In this architecture, the optical fiber acts as a photonic actuator enabling light delivery and localized heating, while drug release is mediated by thermoresponsive microgels immobilized on its surface. The platform is designed for temporary, minimally invasive interventional procedures in which the fiber is introduced through medical instruments such as needles or catheters and removed after treatment. Under these conditions, conventional silica optical fibers provide high optical transmission efficiency, thermal stability, and mechanical robustness. Nevertheless, recent advances in hydrogel-based optical fibers^81,82^ highlight the potential of soft photonic waveguides with improved mechanical compatibility with biological tissues, making them attractive for long-term implantation or biointegrated photonic interfaces and representing a promising direction for future developments.

Within this framework, the FO-LTDR platform represents a versatile approach to localized chemotherapy, combining precise optical actuation with controllable drug delivery. Future work will focus on further engineering optimization and in vivo validation to enable translation toward image-guided and potentially automated therapeutic interventions. Such advances could support minimally invasive, spatially controlled treatment strategies capable of addressing the complexity and heterogeneity of solid tumors.

## Supplementary Information

Supplementary Information.

## Data Availability

The datasets used and/or analysed during the current study available from the corresponding author on reasonable request.
