# Supplementary material for: Light-triggered drug release via fiber optic heater-integrated with thermoresponsive microgels for locoregional cancer therapy
Source: Sci Rep. 2026 Apr 27;16:19407. doi: 10.1038/s41598-026-48134-w (PMC13287653; doi:10.1038/s41598-026-48134-w)
Supplement: Supplementary file 1 — Supplementary Information. [file 41598_2026_48134_MOESM1_ESM.docx]

**Supplementary Information**

**Light-Triggered Drug Release via Fiber Optic Heater-Integrated with Thermoresponsive Microgels for Locoregional Cancer Therapy**

**Tania Mariastella Caputo^1§^, Gaia Maria Berruti^1§^, Silvia Vanni^2§^, Angela Maria Cusano^3^, Claudia Cocchi^2^, Chiara Liverani^2^*, Marco Consales^1^*, Laura Mercatali^4^, Toni Ibrahim^4^, Alessandro De Vita^2#^, Anna Aliberti^1,3#^, Andrea Cusano^1,3#^**

1. Optoelectronics Group, Department of Engineering, University of Sannio, Palazzo Dell' Aquila Bosco Lucarelli, Benevento, Italy.
2. Preclinic and Osteoncology Unit, Bioscience Laboratory, IRCCS Istituto Romagnolo per lo Studio dei Tumori (IRST) “Dino Amadori”, Meldola, Italy
3. CeRICTscrl Regional Center Information Communication Technology, Benevento, Italy
4. Osteoncology, Bone and Soft Tissue Sarcomas and Innovative Therapies Unit, IRCCS Istituto Ortopedico Rizzoli, Bologna, Italy

**Table S1.** MGs monomers quantitative and time of reaction used for each formulation.

|  | NIPAM (g) | NIPMAM (g) | MAAC (g) | BIS (g) | KPS(g) | Time (h) |
| --- | --- | --- | --- | --- | --- | --- |
| DD1 | 0.900 | - | 0.048 | 0.050 | 0.050 | 4 |
| DD2 | - | 0.612 | 0.100 | 0.060 | 0.054 | 5 |
| DD3 | 0.700 | - | 0.100 | 0.026 | 0.030 | 5 |

**Figure S1**. Core offset fusion splice fabrication: a) fibers alignment; b) Final core offset of 6 µm.

**
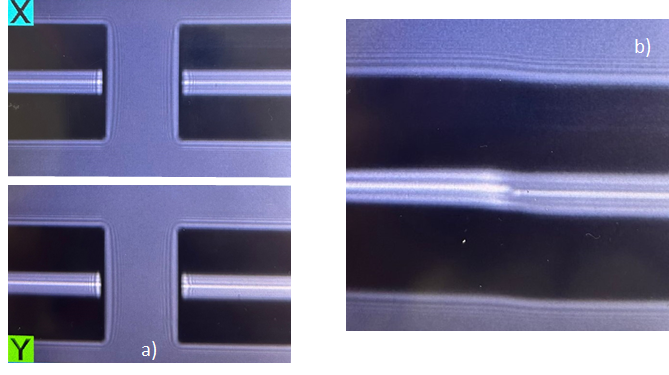
**

B

A


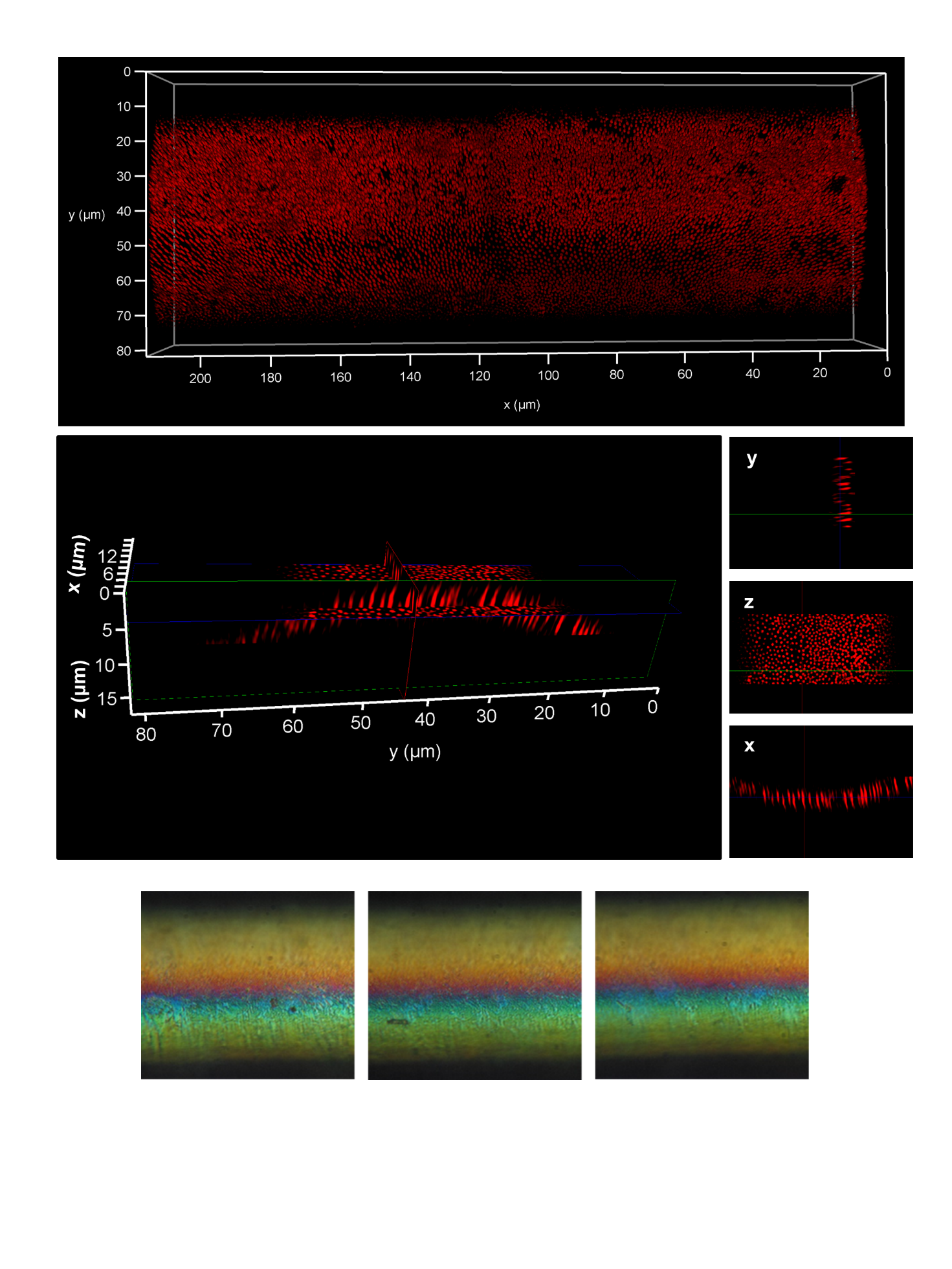
**Figure S2**. CLSM (A) and optical microscope images (B) of DOX@MGs integrated on the FOH gold surface.

**Figure S3**. Typical response of the optical fiber platform during the internal activation test (l= 10 mm and P_IN_ = 210 mW)

**Figure S4**. Efficacy of free DOX on cell proliferation inhibition in MCF7 cell line after 72 h of treatment in 2D and 3D culture models.

**
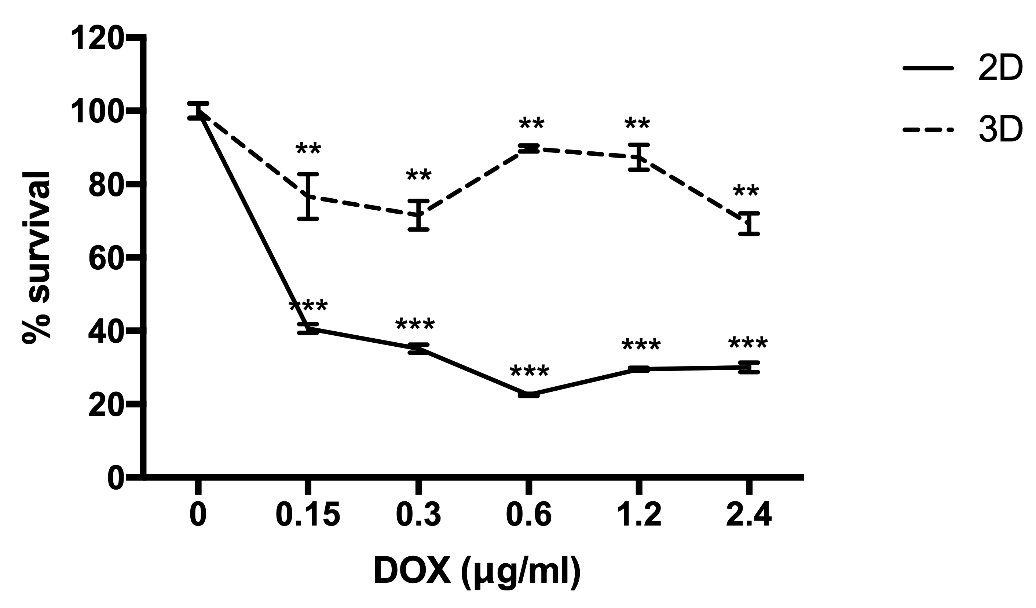
**

**Figure S5**. Cell cycle analysis of the MCF7 cell line treated with DOX after 24h, 48h, or 72h.

**
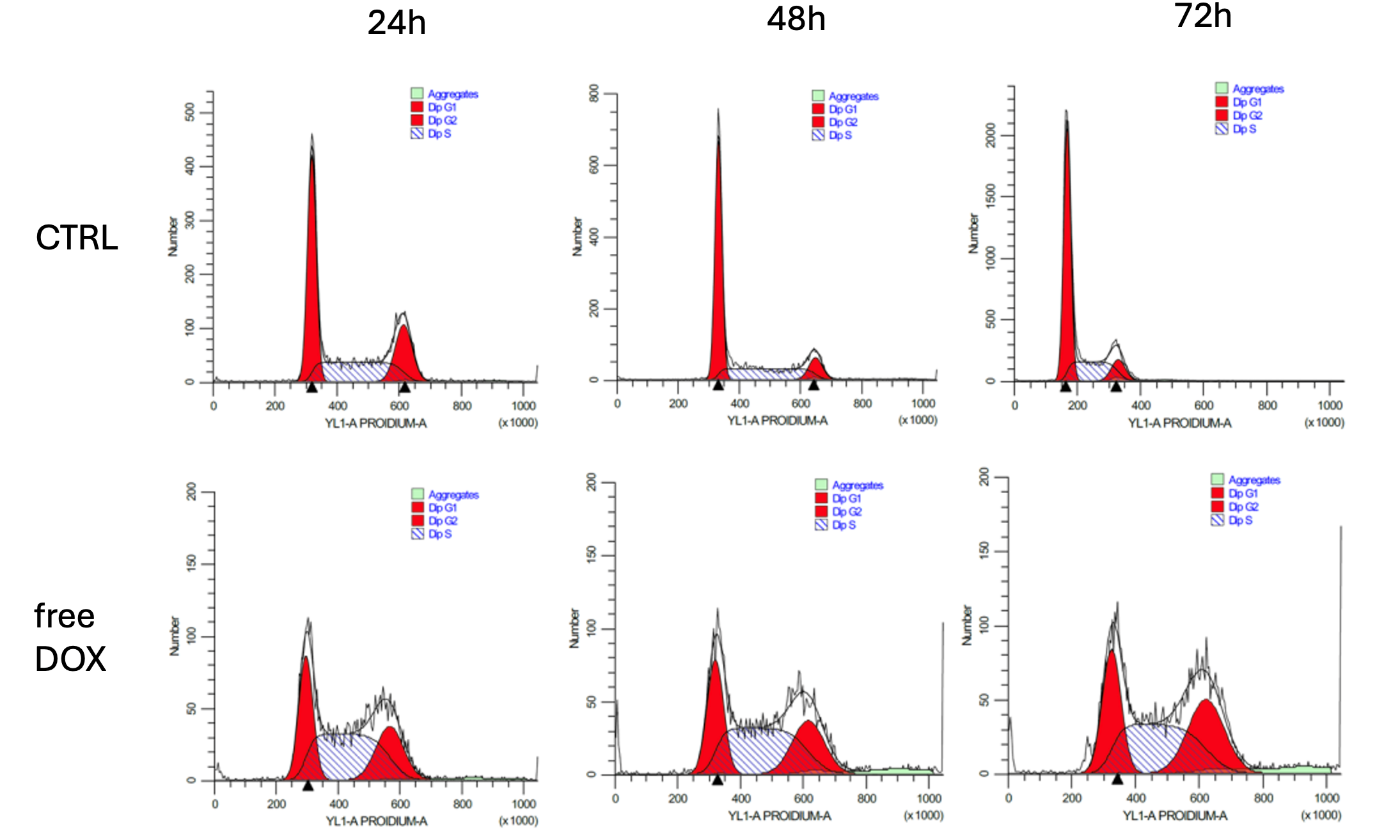
**
